# The complexity of the population dynamics of *Triatoma brasiliensis* in rural north-east Brazil indicated by genetic characterisation

**DOI:** 10.1590/0074-02760250076

**Published:** 2026-03-30

**Authors:** Luiz Osvaldo Rodrigues Silva, Carlota Josefovicz Belisário, Flávio Campos Ferreira, Jorg Heukelbach, Liléia Diotaiuti, Claudia Mendonça Bezerra

**Affiliations:** 1 1Universidade Federal do Ceará, Fortaleza, CE, Brasil; 2 2Fundação Oswaldo Cruz-Fiocruz, Instituto René Rachou, Belo Horizonte, MG, Brasil; 3 3Secretaria Estadual da Saúde do Ceará, Fortaleza, CE, Brasil

**Keywords:** Chagas disease, Triatominae, Triatoma brasiliensis, microsatellites, genetic variability, Ceará

## Abstract

**BACKGROUND:**

*Triatoma brasiliensis*, the primary Chagas disease (CD) vector in the north-east of Brazil, poses a significant challenge for control due to its adaptability and ability to colonise anthropic environments. The limited number of previous studies on the population dynamics of *T. brasiliensis* hinders the development of effective control strategies.

**OBJECTIVES:**

This study characterises the genetic variability of *T. brasiliensis* populations in Jaguaruana using microsatellite markers, in order to understand the population processes of triatomine infestation and reinfestation.

**METHODS:**

We analysed the genetic structure of 229 *T. brasiliensis* specimens collected in the municipality of Jaguaruana in the north-east Brazilian State of Ceará using microsatellite markers.

**FINDINGS:**

Hardy-Weinberg disequilibrium prevailed, with substantial genetic variability (67.2%) among individuals and inbreeding, but genetic differentiation lacked correlation with geographical distance (Mantel’s test).

**MAIN CONCLUSIONS:**

The complex population dynamics in Jaguaruana revealed diverse sources of anthropogenic colonisation, impacting regional control. This study underscores the necessity of comprehending intricate infestation processes for planning effective vector surveillance and control strategies.

Chagas disease (CD) or American trypanosomiasis is caused by the protozoan parasite *Trypanosoma cruzi*, which infects human and non-human animal hosts, through oral transmission, contact with the infected faeces of triatomines and vertical transmission from mother-to-offspring.

According to the World Health Organization (WHO), CD is considered a neglected tropical disease.^1^ CD is endemic in 21 countries in the Americas, and it is estimated that around 6 million people worldwide are infected with *T. cruzi*, with 75 million people living in areas at risk of infection.^1^ In Brazil, there are between 1.9 and 4.6 million people infected, with the majority suffering from the chronic form of CD.^2^ ^,^ ^3^

In Brazil, 64 species of triatomines have been recorded.^4^ Of these, 23% (15) are present in the North-East region of the country, with four species found predominantly inside human dwellings, highlighting the ability of these vectors to establish colonies in human habitations.^5^ ^,^ ^6^ ^,^ ^7^

*Triatoma brasiliensis brasiliensis* (Neiva, 1911) is the most important vector in the Brazilian North-East region.^8^ ^,^ ^9^ ^,^ ^10^ ^,^ ^11^ *Triatoma b. brasiliensis* is a rupestrian subspecies, and in its natural ecotope is mainly associated with rodents, marsupials, and bats.^10^ ^,^ ^12^ ^,^ ^13^ This triatomine subspecies is capable of invading and colonising domiciles and diverse peridomiciliary ecotopes. *Triatoma b. brasiliensis* exhibits a highly eclectic diet, is aggressive, opportunistic, and has significant rates of *T. cruzi* infection. These characteristics make *Triatoma b. brasiliensis* the primary vector of *T. cruzi* transmission within the Caatinga region of the north-east of Brazil.^9^ ^,^ ^14^ ^,^ ^15^

In the municipality of Jaguaruana in the State of Ceará, CD vector control was implemented in the 1970s. About two decades ago, a seroprevalence study^16^ revealed a seropositivity rate of 3.1%, including children under 10 years of age and patients with cardiovascular or digestive symptoms. *T. cruzi*-infected triatomines have also been found in rural localities of Jaguaruana.^17^ Today, in this region, there are still significant home infestations with triatomines, which may be related to several factors previously described in the literature, such as the varied sources of wild and peridomiciliary infestation, the complexity of domestic shelters, operational failures in chemical vector control activities, and the resistance of triatomine populations to the insecticides used in vector control.^8^ ^,^ ^10^ ^,^ ^11^ ^,^ ^12^

In Tauá, another municipality in the State of Ceará, with a different natural ecotope from Jaguaruana (Fig. 1), a previous population genetic study using microsatellites was able to show that *T. brasiliensis* had a panmictic population structure, and, therefore, required intense entomological surveillance in order to control the early reestablishment of infestation foci after the application of insecticide control measures.^18^ In the State of Paraíba, also located in north-eastern Brazil, Almeida et al.^19^ used the mitochondrial *cytb* gene and, in contrast to the study described above, suggested that *T. brasiliensis* populations are genetically structured. Furthermore, these authors observed that reinfestation of anthropic and/or disturbed natural environments is by triatomine individuals from distinct populations. Another study using microsatellites conducted in the State of Rio Grande do Norte, also in north-eastern Brazil, demonstrated gene flow between the distinct populations of *T. brasiliensis* found in sylvatic environments and those from anthropic and/or disturbed natural ecotopes.^20^ Yet another study, undertaken in Currais Novos, a municipality in Rio Grande do Norte, where sequencing of the *cytb* gene revealed four mitochondrial clusters within the 13 sampled *T. brasiliensis* populations. In the same study, analysis of single nucleotide polymorphisms (SNPs) indicated, at most, only very low levels of population genetic structuring suggestive of very high levels of gene flow, if not panmixia.^21^

**Fig. 1:**
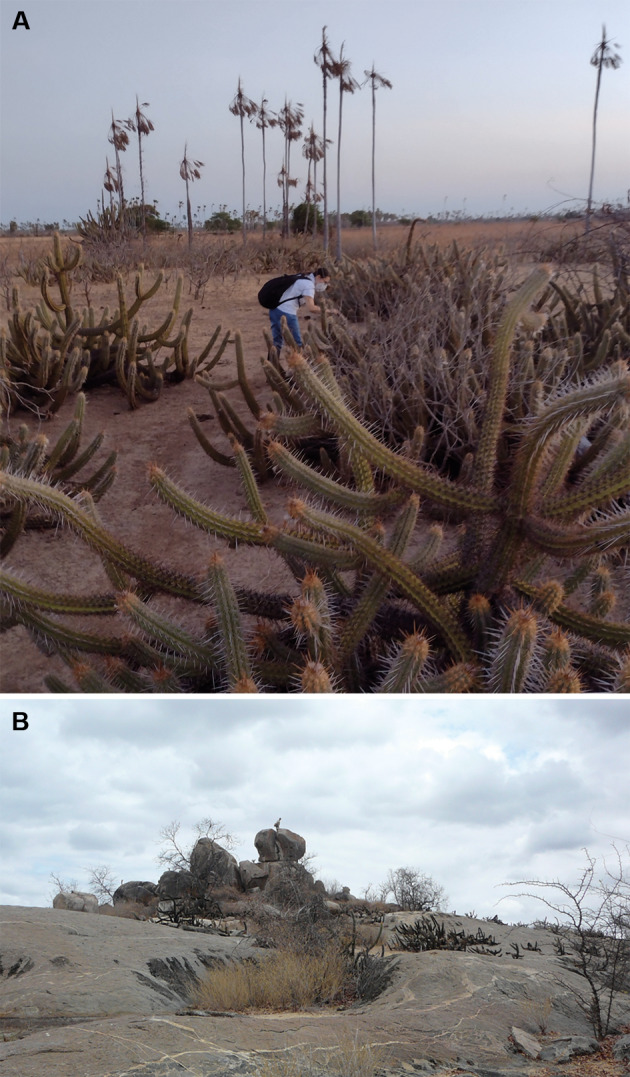
main sylvatic ecotopes of *Triatoma brasiliensis* in Ceará State. (A) In Jaguaruana municipality, the cactus *Pilosocereus gounellei*, which is distributed in discontinuous clumps. (B) In Tauá municipality, large and extensive granite formations.

In general, studies on the variability of microsatellite alleles are useful for understanding population genetic structure, taxonomy, and genomic mapping. Such studies can provide information on gene flow between populations, vector dispersal, and the taxonomic assessment of vectors.^20^-^33^ Microsatellite markers have been shown to be a good tool for investigating the dynamics of triatomine populations, and the design and implementation of new vector control strategies.^20^-^33^ Knowledge about genetic processes and gene flow between environments can elucidate the process of infestation and reinfestation of households by autochthonous triatomines, such as *T. brasiliensis*, which is adapted to both sylvatic and domestic environments.^18^ ^,^ ^20^

This current study characterises the genetic variability of *T. brasiliensis* populations in Jaguaruana using microsatellite markers, in order to evaluate the extent of gene flow between triatomines in this region and further demonstrate that such population genetic analysis is a useful tool for understanding the population processes of triatomine infestation and reinfestation.

## MATERIALS AND METHODS

**Fig. 2:**
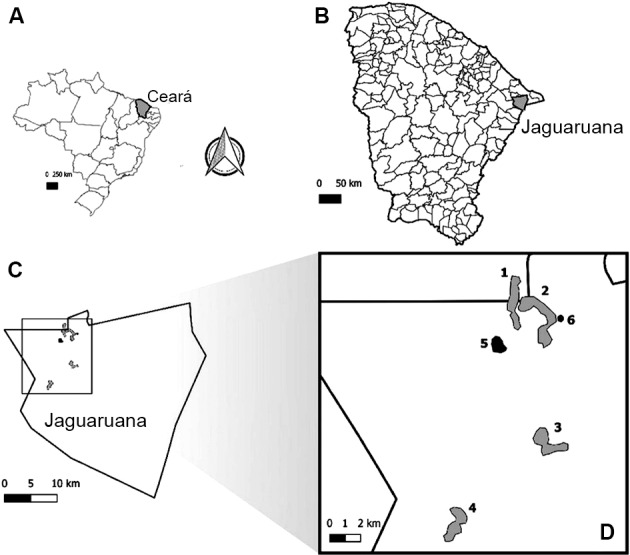
study area. (A) State of Ceará, Brazil; (B) Municipality of Jaguaruana; (C) Localities included in Jaguaruana; (D) Study localities. Gray polygons: areas with collection on compounds: 1: Latadas; 2: Cipriano Lopes; 3: Jenipapeiro; 4: Quixabinha; black polygons: areas with sylvatic collection; 5: João Duarte; 6: Cipriano Lopes.

*Study area* - This study was conducted in the municipality of Jaguaruana, an arid region in the Caatinga in the State of Ceará in the North-East region of Brazil (Fig. 2). Jaguaruana is in the Jaguaribe mesoregion (4º50’02”S; 37º46’52”W), at an altitude of 20 metres above sea level (asl) and 150 km from the capital city of the state, Fortaleza. The climate is mild semi-arid warm tropical, with an average temperature between 26ºC and 28ºC, an average rainfall of 753 mm^3^, and a rainy season from January to April.^33^ The study area has a mix of vast carnaúba palm forests [*Copernicia prunifera* (Mill.) H. E. Moore] and desert areas with xerophytic vegetation, shrubby and spiny, where the xique-xique cactus (*Pilosocereus gounellei*, F.A.C. Weber) is abundant and commonly serves as a shelter for small rodents and reptiles.^34^

*Triatoma brasiliensis* is found in practically the entire municipality, both in sylvatic and anthropic environments. The present study included rural locations with a history of *T. brasiliensis* infestation, which also regularly implemented insecticide-based vector control, namely: Latadas [44 domiciliary units (DUs)], Cipriano Lopes (36 DUs), Jenipapeiro (36 DUs) and Quixabinha (28 DUs) (Fig. 2). A DU usually consists of both the intradomicile (human habitation) and the peridomicile (surroundings of the human dwelling), including fences, animal shelters, piles of objects (tiles, bricks, stones, wood, etc.), as well as permanent and temporary constructions. The ecological complexity and stability of the peridomestic ecotope is responsible for maintaining populations of triatomines, where availability of shelter, food sources, and relatively constant abiotic conditions favour colonisation and high population densities. Sylvatic vector samples were also collected from five rocky outcroppings in the locality of João Duarte, and from a cluster of xique-xique in Cipriano Lopes (Fig. 2).

*Collection of Triatomine samples* - The triatomines were captured manually and exhaustively in the DUs by endemic disease agents from the municipality of Jaguaruana, following the recommended standard procedures,^35^ ^,^ ^36^ in locations monitored by the State Health Department in October 2021. DUs with the presence of triatomines (intradomiciliary and/or peridomiciliary) were sprayed with alpha-cypermethrin SC 20% (Fersol Industria e Comércio). The triatomines collected were identified according to DU and ecotope of origin. For the population genetic analysis, 252 insects were used, pooled into 29 separate samples. We also analysed other frozen triatomines that were collected in the same locations between 2016 and 2018 and were provided by REMOT (Monitoring Network for the Susceptibility of Brazilian Triatomine Populations to Insecticides). The individual triatomines were pooled into samples according to their collection site (*i.e.*, individual DU or sylvatic environment). When triatomines were collected from more than one ecotope within the same DU, separate pooled samples were made for each different ecotope, while triatomines captured from different sites within the same sylvatic location were also pooled separately (Table I). Each pooled sample was composed of a minimum of five individuals as required for analysis of molecular variance (AMOVA).^36^ ^,^ ^37^

**TABLE I:** Number of *Triatoma brasiliensis* insects captured, by location, ecotope and year in the municipality of Jaguaruana in the State of Ceará, Brazil

Location of collection	Sample abbreviation	Domiciliary unit	Intra/Peri/Sylvatic	Ecotope	Year of collection	Southern latitude	West longitude	Number of insects
Latada	Lat18c1	NI	Peri	NI	2016	-4.7738593	-37.8306312	9
	Lat23	NI	Peri	NI	2018	-4.7738593	-37.8306312	10
	Lat3c1	18c1	Peri	chicken coop	2021	-4,7637007	-37,831751	10
	Lat11	23	Peri	chicken coop	2021	-4,7599111	-37,8308041	10
	Lat13	3c1	Peri	roof tile	2021	-4.7065176	-37.832823	10
	Lat14c1	13	Peri	wood	2021	-4,7652177	-37,8314035	10
	CLop17	14c1	Peri	chicken coop	2021	-4,7646038	-37,8331039	9
	CLop33c1p1	11	Intra	front porch	2021	-4.7658002	-37.832582	10
Cipriano Lopes	CLop33c1p2	NI	Peri	NI	2016	-4.78018	-37.81823	10
	CLop15c2	NI	Peri	NI	2018	-4.78018	-37.81823	10
	CLop27	33c1	Peri	firewood 1	2021	-4,7657745	-37,8271903	10
	CLop23p1		Peri	firewood 2	2021	-4,7657607	-37,827158	10
	CLop23p2	15c2	Peri	pigsty	2021	-4.7764018	-37.8202555	10
	Quix5	27	Peri	pigsty	2021	-4.7651053	-37.8252401	10
	Jen6	23	Peri	wood	2021	-4,7650495	-37,8222092	9
	Jen1		Peri	chicken coop	2021	-4,7651269	-37,8223627	10
	Jen6c1	17	Intra	front porch	2021	-4.7707044	-37.8161672	7
	Jen15	NI	Sylvatic	carnaúba-xique-xique-floor	2021	-4,7709308	-37,8157584	7
Quixabinha	JDuaWild1	NI	Peri	NI	2016	-4.8555173	-37.8540889	10
	JDuaWild2	5	Peri	chicken coop	2021	-4.8536332	-37.8554654	5
Jenipapeiro	JDuaWild3	6	Peri	chicken coop	2021	-4.82338	-37.8188	7
	JDuaWild4	1	Peri	carnaúba	2021	-4,8259575	-37,8100437	6
	CLopWild	6c1	Peri	chicken coop	2021	-4,8233762	-37,81883	5
	JDuaWild5	15	Peri	pigsty	2021	-4,8227348	-37,8209339	9
João Duarte	LatR25	-	Sylvatic	rock 3	2021	-4,7827741	-37,8389039	11
	LatR70	-	Sylvatic	rock 4	2021	-4,7828295	-37,8393717	8
	CLopR26	-	Sylvatic	rock 5	2021	-4,7825214	-37,8392454	5
	QuixR27	-	Sylvatic	rock 6	2021	-4,7821158	-37,839741	6
	CLopR69	-	Sylvatic	rock Luiz	2021	-4,7811206	-37,8386576	9

NI: Not identified.

*Microsatellite genotyping* - Two legs from each insect were used for genomic DNA extraction using the Wizard® Genomic DNA Purification Kit (Promega) and the protocol of Borges et al.^10^ The DNA was quantified using a NanoDrop 1000 Spectrophotometer (Thermo Scientific) and stored at -20ºC. Primers were tested for nine microsatellite loci for *T. brasiliensis*: Tb728, Tb830, Tb860, Tb7180, Tb8112, Tb8124,^38^ B2146 (GenBank: KT355796.1), B8102 (GenBank: KT355797.1) and B8150 (GenBank KT355795.1). Polymerase chain reactions (PCR) amplifications were carried out using a final volume of 10 μL containing 1 unit of Platinum® Taq DNA polymerase (Invitrogen), 1X buffer, 1.5 mM MgCl2, 1 mM dNTP, 5 pmol for each primer, 2 ng of DNA and ultrapure water. The forward primers were labelled with a bioluminescent probe. The reactions were performed using a Veriti® 96-Well thermal cycler (Thermo Fisher Scientific) with the following cycling conditions: an initial denaturation at 95ºC for 5 min; followed by 28 cycles of 94ºC for 30 s, primer-dependent temperature annealing for 30 s, and extension at 72ºC for 45 s; and then a final extension at 72ºC for 5 min. The annealing temperatures for each locus were: 48ºC for Tb860; 54ºC for Tb8112; 52ºC for Tb 2146; and 56ºC for Tb8102; followed by touchdown (*i.e.*, incremental reduction in the annealing temperature): 60→50ºC and 58ºC for Tb728, Tb830, Tb7180, Tb8124. In order to determine the size of the amplicons, the PCR products were diluted 1:10 in pure water together with a GeneScan™ 500 LIZ® size standard (Thermo Fisher Scientific) and genotyped on an ABI 3730 DNA Analyzer (Applied Biosystem®) at the DNA Sequencing Platform of the René Rachou Institute. The chromatograms were analysed using the Geneious 10.1.2© program (Biomatters Limited).

*Data analysis* - Several analyses were conducted: obtaining the number and size of alleles for each locus, observed (OH) and expected heterozygosity (EH), Hardy-Weinberg equilibrium (HW) verification, AMOVA, calculation of fixation indices (*F*st, *F*is, and *F*it) and the Mantel test (Arlequin version 3.5).^39^ The statistical tests were carried out using a significance level of 5% and a maximum loss of 5% of amplified alleles. Due to the large number of pairwise *F*st comparisons, p-values were corrected using the false discovery rate (FDR) method to control the false positive rate.^40^ A Neighbor-Joining dendrogram was generated with the pairwise *F*st values using POPTREEW.^41^ It is important to emphasise that the primary purpose of the tree is to make it easier to visualise the relationships obtained by the pairwise *F*st. Only bootstrap values greater than 50% are statistically supported and were arbitrarily rooted because no outgroup was used. Hardy-Weinberg equilibrium deviations were evaluated in Genepop v4.3 using the Guo and Ye exact test. The Markov chain procedure was conducted with 10,000 steps, 20 independent replicates, and 5,000 iterationsper replicate.^42^ ^,^ ^43^ Allelic richness was calculated using the rarefaction statistical method based on a minimum of ight genes per sample (HP-Rare version 1.1).^44^ ^,^ ^45^ The presence of null alleles was checked (Micro-Checker 2.2.3)^46^ and their influence was assessed using the null allelic exclusion (NAE) methodology with a 95% confidence interval (CI) generated using 10,000 bootstraps (FreeNa).^47^ The first-generation migrant test was carried out to identify potential immigrants within each sample and their most likely origin.^48^ The test was performed using the computational criterion of the frequency-based method proposed by Paetkau^49^ together with the algorithm described by Paetkau.^48^ This analysis was carried out using a total of 10,000 Monte Carlo chains for each individual simulation with a p ≤ 0.05 for each result generated (GENECLASS2).^50^ In order to study the population genetic structure of the *T. brasiliensis* sampled, one to 15 genetic clusters (*K*) were evaluated using a total of 20 repetitions and 1,000,000 iterations per Markov and Monte Carlo Chain (burn-in 100,000) for each K evaluated with the correlated allele frequencies between populations (Structure version 2.3.4).^51^ ^,^ ^52^ The best *K* value was identified using the metrics MedMedK, MedMeanK, MaxMedK, and MaxMeanK^53^ ranging from 1 to 10 (Structure Selector^54^).

*Ethics* - This study was approved by the Human Research Ethics Committee of the Federal University of Ceará (UFC), under license no. 6.024.559 on 26 April 2023.

## RESULTS

Of the nine primer pairs tested, only seven had amplifications, and only five of these amplified microsatellite loci were polymorphic (Table II). For the five polymorphic microsatellite loci, the average number of alleles (NA) observed per locus ranged from 1.9 (Tb860) to 5.1 (Tb7180), with an overall average of 3.2. Sample 1 from João Duarte Silvestre (JDuaWild1) had both the highest average allelic number (NA = 4.2) and allelic richness (AR = 3.3), while DU 18c1 from Latada (Lat18c1) had the lowest average NA (= 2.0) and AR (= 1.8). The NA and AR of all the samples are shown in Table III. The size of each individual allele is given in Supplementary data (Table I).

**TABLE II:** *Triatoma brasiliensis* microsatellite loci amplified from samples captured form different rural localities in the municipality of Jaguaruana in the State of Ceará, Brazil

Name		Sequence (5’- 3’)	Motif	Annealing temperature	Expected size(38)	Observed size
Tb728	F:	CTACAGCGATTTGTCTCG-NED	(GT)2AT(GT)12	58ºC	306 - 316	308 - 316
	R:	TATTGCATCATGTTTATTGG				
Tb830	F:	TGTCAGATGCATGGTGATAC-6FAM	(AC)15	58ºC	274 - 298	276 - 290
	R:	CATGGAAGATACCTAAACGG				
Tb860	F:	CGTTTTAGTAAGGAATGG-PET	(CT)5 (CA)10(CTCA)3	48ºC	392 - 396	394
	R:	ATTGTGCCAAAATCAGGT				
Tb7180	F:	TGACCTACCGCCACATTAC-VIC	(CATA)3(CA)8 TA(CA)18(GA)3	58ºC	220 - 246	214 - 246
	R:	CAAATTTTCGATACCGCGATAG				
Tb8124	F:	GCCACTGTGTTCTCATTCC-NED	(CA)18	58ºC	218 - 246	224 - 242
	R:	TGGTGTGATGCTCAGAAGG				

**TABLE III:** Number of alleles (NA) and allelic richness (AR) per microsatellite locus for *Triatoma brasiliensis* samples collected from the municipality of Jaguaruana in the State of Ceará, Brazil

Sample\Locus	Tb728		Tb830		Tb860		Tb7180		Tb8124		Average	
	NA	AR	NA	AR	NA	AR	NA	AR	NA	AR	NA	AR
Lat18c1	2	1.8	1	1.0	2	2.0	3	2.1	2	2.0	2.0	1.8
Lat23	2	1.9	3	2.7	1	1.0	5	3.3	2	1.6	2.6	2.1
Lat3c1	2	2.0	3	2.4	2	1.6	5	4.0	5	3.8	3.4	2.7
Lat11	4	3.5	3	2.4	2	1.4	4	3.2	5	4.0	3.6	2.9
Lat13	3	2.0	4	3.0	2	1.9	5	3.6	4	3.0	3.6	2.7
Lat14c1	4	3.4	3	2.6	2	1.9	5	4.0	5	3.9	3.8	3.2
CLop17	3	2.4	3	2.8	2	2.0	4	3.5	4	3.2	3.2	2.8
CLop33c1p1	2	1.9	2	1.9	2	2.0	5	3.9	2	1.6	2.6	2.3
CLop33c1p2	4	2.7	3	2.6	2	1.8	6	4.2	4	3.0	3.8	2.8
CLop15c2	3	2.8	2	1.9	2	1.4	6	4.0	2	1.9	3.0	2.4
CLop27	3	2.8	4	2.9	2	1.9	6	4.4	4	3.0	3.8	3.0
CLop23p1	2	1.8	4	3.4	2	1.8	6	4.4	3	2.6	3.4	2.8
CLop23p2	2	1.4	4	3.5	1	1.0	6	3.8	4	3.2	3.4	2.6
Quix5	2	2.0	3	3.0	1	1.0	3	3.0	5	4.6	2.8	2.7
Jen6	2	2.0	2	2.0	2	2.0	3	2.1	3	2.7	2.4	2.2
Jen1	2	2.0	3	2.6	2	2.0	5	4.2	2	2.0	2.8	2.6
Jen6c1	2	2.0	2	2.0	2	2.0	4	3.8	1	1.0	2.2	2.2
Jen15	3	2.4	3	2.7	2	2.0	6	4.0	3	2.4	3.4	2.7
JDuaWild1	4	3.7	3	2.9	3	2.2	6	4.1	5	3.4	4.2	3.3
JDuaWild2	4	3.4	2	2.0	1	1.0	7	5.2	3	2.9	3.4	2.9
JDuaWild3	4	3.6	2	2.0	2	2.0	5	4.6	2	2.0	3.0	2.8
JDuaWild4	3	2.9	2	2.0	3	2.3	5	4.6	4	3.9	3.4	3.1
CLopWild	3	2.4	2	1.9	2	2.0	5	3.8	3	2.8	3.0	2.6
JDuaWild5	4	3.5	3	2.4	2	1.6	7	5.2	3	2.7	3.8	3.1
LatR25	3	2.4	3	2.8	2	1.8	6	4.4	3	2.9	3.4	2.9
LatR70	3	2.2	3	2.4	2	2.0	5	3.9	2	2.0	3.0	2.5
CLopR26	2	1.9	2	1.8	2	1.9	4	3.0	4	2.9	2.8	2.3
QuixR27	3	2.2	2	2.0	2	1.9	6	4.4	4	3.0	3.4	2.7
CLopR69	3	2.5	3	2.5	1	1.0	6	4.9	4	3.2	3.4	2.8
Average	2.9	2.5	2.7	2.4	1.9	1.7	5.1	3.9	3.3	2.8	3.2	2.7
Total NA	4		4		3		14		11			

Samples (locality name, domiciliary unit identification). R: Remot; Wild: wild ecotope); Lat: Latadas; Clop: Cipriano Lopes; Quix: Quixabinha; Jen: Jenipapeiro; JDua: João Duarte.

The locus with the lowest average OH was Tb8124 (0.16), and the highest was Tb7180 (0.56). As for the average EH, Tb860 had the lowest average (0.31), and Tb8124 had the highest (0.65), respectively. Loci Tb728 and Tb830 were in Hardy-Weinberg equilibrium (HW). Regarding populations, most showed HW disequilibrium due to an excess of homozygotes (p-values ≤ 0.05 for the heterozygote deficit test), except for CLopR69. The results of OH, EH and HW for the samples are detailed in Table IV.

**TABLE IV:** Values of observed heterozygosity (OH), expected heterozygosity (EH), and Hardy-Weinberg equilibrium (HW) for each locus in *Triatoma brasiliensis* samples collected from Jaguaruana, Ceará, Brazil

Sample\Locus		Tb728	Tb830	Tb860	Tb7180	Tb8124	HW p-value
Lat18c1	OH	0.30	0.40	0.00	0.11	0.00*	0.0026*
	EH	0.27	0.44	0.00	0.31	0.44*	
Lat23	OH	0.40	0.30*	0.00	0.70	0.00*	0.0477*
	EH	0.34	0.58*	0.00	0.62	0.19*	
Lat3c1	OH	0.33	0.80	0.20	0.60	0.30*	0.0086*
	EH	0.42	0.54	0.19	0.78	0.71*	
Lat11	OH	0.70	0.70	0.10	0.60	0.70*	0.4404
	EH	0.72	0.57	0.10	0.64	0.79*	
Lat13	OH	0.30	0.40*	0.40	0.20*	0.10*	0.0001*
	EH	0.28	0.66*	0.34	0.72*	0.59*	
Lat14c1	OH	0.67	0.44	0.22	0.44*	0.33*	0.0005*
	EH	0.73	0.52	0.37	0.79*	0.75*	
CLop17	OH	0.43	0.71	0.57	0.14*	0.43	0.0314*
	EH	0.38	0.62	0.44	0.74*	0.58	
CLop33c1p1	OH	0.50	0.00*	0.70	0.50*	0.00*	0.0016*
	EH	0.39	0.34*	0.48	0.77*	0.19*	
CLop33c1p2	OH	0.40	0.10*	0.30	0.70	0.20*	0.0000*
	EH	0.44	0.53*	0.27	0.76	0.51*	
CLop15c2	OH	0.80	0.40	0.10	0.40	0.20	0.0128*
	EH	0.63	0.34	0.10	0.72	0.34	
CLop27	OH	0.50	0.40	0.50	1.00	0.20*	0.1100
	EH	0.64	0.55	0.39	0.81	0.63*	
CLop23p1	OH	0.25	0.38*	0.25	0.63	0.00*	0.0008*
	EH	0.23	0.69*	0.23	0.78	0.52*	
CLop23p2	OH	0.10	0.70	0.00	0.40*	0.20*	0.0015*
	EH	0.10	0.74	0.00	0.71*	0.61*	
Quix5	OH	0.25	0.50	0.00	1.00	0.80	0.3424
	EH	0.25	0.68	0.00	0.71	0.82	
Jen6	OH	0.57	0.00*	0.57	0.14	0.00*	0.0006*
	EH	0.44	0.53*	0.53	0.27	0.48*	
Jen1	OH	0.50	0.67	1.00	0.83	0.00*	0.4950
	EH	0.41	0.53	0.55	0.80	0.48*	
Jen6c1	OH	0.60	0.00*	0.60	0.80	0.00	0.2788
	EH	0.56	0.53*	0.47	0.78	0.00	
Jen15	OH	0.67	0.56	0.33	0.56	0.11*	0.0400*
	EH	0.58	0.57	0.42	0.76	0.50*	
JDuaWild1	OH	0.73	0.64*	0.45	0.55	0.18*	0.0022*
	EH	0.77	0.68*	0.39	0.77	0.63*	
JDuaWild2	OH	1.00	0.38	0.00	0.88*	0.13*	0.0953
	EH	0.71	0.53	0.00	0.88*	0.63*	
JDuaWild3	OH	0.60	0.75	0.25	0.60	0.00	0.1477
	EH	0.64	0.54	0.25	0.82	0.43	
JDuaWild4	OH	0.67	0.40	0.33	0.40*	0.17*	0.0003*
	EH	0.67	0.53	0.32	0.82*	0.80*	
CLopWild	OH	0.43	0.43	0.71	0.71	0.00*	0.1075
	EH	0.38	0.36	0.49	0.67	0.66*	
JDuaWild5	OH	0.33*	0.56	0.14	0.44*	0.22*	0.0000*
	EH	0.70*	0.57	0.14	0.87*	0.63*	
LatR25	OH	0.33	0.56	0.00	0.67	0.00*	0.0001*
	EH	0.45	0.66	0.23	0.81	0.68*	
LatR70	OH	0.40	0.40	0.50	0.60	0.00*	0.0029*
	EH	0.35	0.56	0.48	0.76	0.53*	
CLopR26	OH	0.40	0.33	0.33	1.00*	0.10*	0.1851
	EH	0.34	0.29	0.30	0.66*	0.50*	
QuixR27	OH	0.20	0.40	0.50	0.50*	0.10*	0.0000*
	EH	0.35	0.53	0.39	0.81*	0.65*	
CLopR69	OH	0.40	0.20*	0.00	0.50*	0.20*	0.0000*
	EH	0.48	0.48*	0.00	0.86*	0.61*	
Average	OH	0.47	0.41*	0.32	0.56*	0.16*	
	EH	0.51	0.61*	0.31	0.81*	0.65*	

*p ≤ 0.05. Samples (locality name, domiciliary unit identification). R: Remot; Wild: wild ecotope; Lat: Latadas; Clop: Cipriano Lopes; Quix: Quixabinha; Jen: Jenipapeiro; JDua: João Duarte.

AMOVA showed that 67.2% of the genetic variability is among all individuals analysed, 22.6% among individuals from the same sample and 10.2% among samples. The fixation indices showed a significant p-value ≤ 0.05 (Table V). The inbreeding coefficient (*F*is) ranged from -0.09 (Jenipapeiro DU 1 and Cipriano Lopes DU 26 from REMOT) to 0.48 CLopR69. Positive *F*is values were observed in the following samples: Latadas DUs 23, 3c1, 11; Cipriano Lopes DUs 17, 33c1p1, 15c2, and sylvatic habitat (CLopWild); Jenipapeiro DU 6c1; João Duarte sylvatic habitats 2 and 3 (JDuaWild2 and JDuaWild3). The samples Quixabinha DU 5, Cipriano Lopes 26 from REMOT and Jenipapeiro DUs 1 showed negative *F*st values. The population differentiation index (pairwise *F*st) ranged from 0 to 0.44. The comparisons with the lowest values were: Cipriano Lopes wild (CLopWild) with Latadas DU 14c1; João Duarte wild environments 2 and 5 (JDuaWild2 and JDuaWild5); Quixabinha DU27 from REMOT with João Duarte wild ecotope 4 (JDuaWild4); Latadas DU25 and DU70 from REMOT; and Latadas DU 70 from REMOT with João Duarte wild 5 (JDuaWild5). The most differentiated samples were Jenipapeiro DU 6c1 and Latadas DU 18c1. Negative *F*st indices were considered indicative of no genetic differentiation (Table VI). Mantel’s test did not indicate a correlation between genetic differentiation and geographical distance.

**TABLE V:** Analysis of molecular variance (AMOVA) and the fixation index for *Triatoma brasiliensis* collected from the municipality of Jaguaruana in the State of Ceará, Brazil

Variation source	Variation’s components	Variation’s percentage	Fixation index
Between populations	0.14(Va)	10.24	0.10* (*F*st)
Between individuals within populations	0.31 (Vb)	22.57	0.25* (*F*is)
Between individuals	0.94 (Vc)	67.19	0.33* (*F*it)

**Fig. 3:**
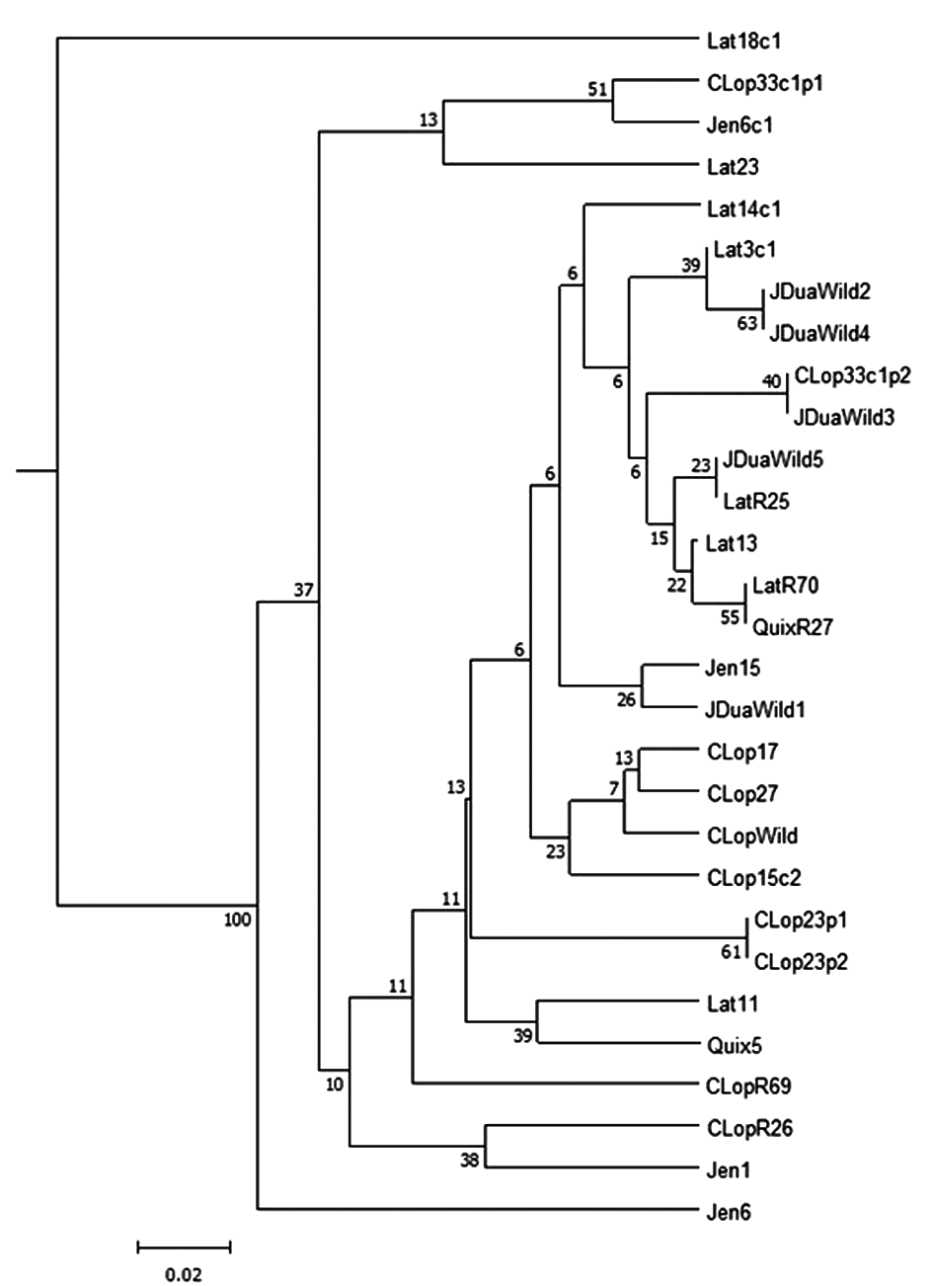
dendogram Neighbour joing of Fst pairwise of *Triatoma brasiliensis* from Jaguaruana, Ceará. Samples (locality name, domiciliary unit identification). R: Remot; Wild: wild ecotope; Lat: Latadas; Clop: Cipriano Lopes; Quix: Quixabinha; Jen: Jenipapeiro; JDua: João Duarte.

**Fig. 4:**
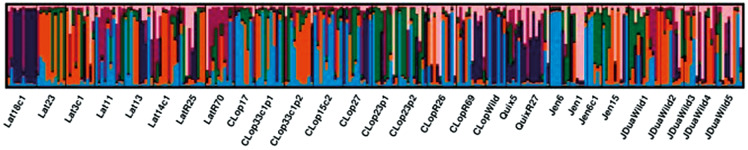
bar chart representing genetic diversity for *Triatoma brasiliensis* from Jaguaruana, Ceará. Each bar represents an individual, and each colour represents one of the six clusters. Samples (locality name, domiciliary unit identification). R: Remot; Wild: wild ecotope; Lat: Latadas; Clop: Cipriano Lopes; Quix: Quixabinha; Jen: Jenipapeiro; JDua: João Duarte.

The genetic structure of the analysed populations was assessed using a neighbour-joining (NJ) tree based on the pairwise *F*st index (Fig. 3) and a Bayesian clustering analysis performed using STRUCTURE (Fig. 4). In the dendrogram (Fig. 3), the sylvatic populations of João Duarte (JDuaWild2 and JDuaWild4) formed a single cluster, while the other populations from the same locality did not cluster with the first two. There is also genetic similarity between the populations Latadas REMOT (LatR70) and Quixabinha REMOT (QuixR27). The Latadas population (Lat18c1) was the most differentiated. The dendrogram showed the two peridomiciliary annexes of Cristiano Lopes’ DU 23 (CLop23p1 and CLop23p2) clustered. A different situation occurred in the peridomiciliary annexes of DU 33 in the same locality (CLop33p1 and CLop33p2). Three of the four statistics evaluated supported supported K = 6 as the most likely number of clusters [Supplementary data (Figure)]. The genetic structure analysis indicated clusters with high diversity in 18 of the 20 runs performed. The most homogeneous samples were those from Latadas DU 18c1 and DU 23, which did not resemble each other.

The presence of null alleles was observed for each of the five polymorphic loci. However, this did not influence the *F*st analyses, since the either excluding null alleles (0.105) or including them (0.102) were within the confidence interval of the NAE method (that excludes null alleles) (0.088 to 0.124 without null alleles; 0.091 to 0.118 with null alleles) [Supplementary data (Tables II-IV)]. The test of the first generation of migrants detected 19 individuals, which are shown in Table VI. Quixabinha DU 5 was the only sample of origin that had two individuals reclassified. Samples from Latadas DU 18c1, Jenipapeiro DU 15 and João Duarte ecotope wild 3 (JDuaWild3) received two individuals; Latadas DU 70 from REMOT received three individuals (Table VI).

**TABLE VI:** Geographic distances between the sampling locations in kilometres (above the diagonal), pairwise *F*st values (below the diagonal), and *F*is values (on the diagonal) for *Triatoma brasiliensis* collected from the municipality of Jaguaruana in the State of Ceará, Brazil

Sample	Lat18c1	Lat23	Lat3c1	Lat11	Lat13	Lat14c1	CLop17	CLop33c1p1	CLop33c1p2	CLop15c2	CLop27	CLop23p1	CLop23p2	Quix5	Jen6	Jen1	Jen6c1	Jen15	JDuaWild1	JDuaWild2	JDuaWild3	JDuaWild4	CLopWild	JDuaWild5	LatR25	LatR70	CLopR26	QuixR27	CLopR69
Lat18c1	0.44	0.43	6.37	0.25	0.17	0.18	1.90	0.56	0.56	1.90	0.74	1.07	1.05	10.35	6.80	7.34	6.80	6.68	2.27	2.29	2.25	2.23	1.95	2.09	1.14	2.37	10.52	2.37	1.14
Lat23	0.45*	0.20*	5.95	0.68	0.59	0.58	2.02	0.77	0.77	2.18	0.85	1.11	1.10	10.79	7.19	7.70	7.19	7.08	2.70	2.72	2.69	2.66	2.07	2.52	1.55	2.65	10.95	2.65	1.55
Lat3c1	0.31*	0.14*	0.15*	6.60	6.54	6.47	7.38	6.63	6.62	7.90	6.58	6.62	6.63	16.57	13.10	13.53	13.10	13.00	8.52	8.53	8.49	8.45	7.42	8.33	7.50	8.36	16.75	8.36	7.50
Lat11	0.26*	0.19*	0.08*	0.01*	0.15	0.15	1.90	0.60	0.60	1.81	0.82	1.15	1.14	10.10	6.59	7.15	6.59	6.47	2.02	2.04	2.00	1.98	1.95	1.83	0.92	2.26	10.27	2.26	0.92
Lat13	0.21*	0.15*	0.03	0.07*	0.47	0.20	1.80	0.47	0.47	1.75	0.68	1.02	1.00	10.20	6.62	7.16	6.62	6.51	2.12	2.15	2.11	2.10	1.85	1.94	0.97	2.22	10.36	2.22	0.97
Lat14c1	0.24*	0.19*	0.06	0.06*	0.09	0.35	2.00	0.67	0.67	1.94	0.87	1.21	1.19	10.22	6.73	7.29	6.73	6.61	2.12	2.14	2.11	2.08	2.05	1.94	1.07	2.39	10.38	2.39	1.07
CLop17	0.25*	0.17*	0.09*	0.06	0.02	0.08	0.18*	1.34	1.34	0.78	1.18	0.92	0.93	10.21	5.87	6.19	5.87	5.82	2.86	2.91	2.88	2.91	0.05	2.75	1.64	1.08	10.34	1.08	1.64
CLop33c1p1	0.30*	0.10*	0.17*	0.17*	0.09*	0.14*	0.05	0.23*	0.00	1.41	0.23	0.56	0.54	10.27	6.48	6.96	6.48	6.38	2.30	2.33	2.29	2.29	1.39	2.13	0.98	1.89	10.43	1.89	0.98
CLop33c1p2	0.31*	0.02	0.04	0.10*	0.05	0.06	0.06	0.05	0.33	1.41	0.22	0.55	0.54	10.27	6.48	6.96	6.48	6.38	2.30	2.33	2.30	2.29	1.39	2.13	0.98	1.89	10.43	1.89	0.98
CLop15c2	0.29*	0.16*	0.13*	0.08*	0.07	0.09*	0.04	0.09*	0.09*	0.11*	1.37	1.28	1.28	9.44	5.23	5.63	5.23	5.16	2.19	2.24	2.21	2.25	0.79	2.11	1.19	0.48	9.57	0.48	1.19
CLop27	0.19*	0.14*	0.08*	0.08*	0.05	0.03	0.03	0.08*	0.06	0.04	0.15	0.34	0.32	10.41	6.53	6.98	6.53	6.43	2.48	2.52	2.48	2.48	1.24	2.32	1.14	1.85	10.56	1.85	1.14
CLop23p1	0.33*	0.16*	0.09*	0.12*	0.03	0.14*	0.04	0.12*	0.10*	0.14*	0.08	0.36	0.02	10.53	6.50	6.91	6.50	6.42	2.71	2.75	2.71	2.72	0.97	2.56	1.35	1.74	10.67	1.74	1.35
CLop23p2	0.32*	0.18*	0.08	0.10*	0.02	0.17*	0.05	0.17*	0.12*	0.13*	0.11*	-0.02	0.36	10.51	6.50	6.91	6.50	6.41	2.69	2.73	2.69	2.70	0.98	2.54	1.34	1.74	10.66	1.74	1.34
Quix5	0.28*	0.27*	0.07	0.04	0.07	0.12*	0.09	0.24*	0.13*	0.19*	0.09	0.13*	0.08	-0.22*	5.28	5.91	5.28	5.15	8.10	8.08	8.12	8.15	10.21	8.28	9.30	9.16	0.26	9.16	9.30
Jen6	0.27*	0.31*	0.23*	0.19*	0.12	0.15*	0.08	0.10	0.21*	0.18*	0.12*	0.12	0.18*	0.25*	0.45	1.01	0.00	0.25	5.04	5.06	5.08	5.15	5.85	5.19	5.67	4.81	5.30	4.81	5.67
Jen1	0.30*	0.33*	0.11*	0.19*	0.08	0.16*	0.15*	0.24*	0.18*	0.26*	0.14*	0.16*	0.17*	0.14*	0.24*	-0.09*	1.02	1.26	5.78	5.80	5.82	5.89	6.16	5.91	6.23	5.18	5.89	5.18	6.23
Jen6c1	0.44*	0.13*	0.19*	0.17*	0.13	0.15*	0.12	0.04	0.11	0.14*	0.12*	0.09	0.17*	0.28*	0.12	0.24*	0.16*	0.24	5.04	5.06	5.08	5.15	5.85	5.19	5.67	4.81	5.30	4.81	5.67
Jen15	0.33*	0.19*	0.03	0.09*	0.02	0.09*	0.08	0.14*	0.09*	0.12*	0.08*	0.09	0.10*	0.12*	0.18*	0.04	0.09	0.23	4.87	4.89	4.92	4.98	5.80	5.03	5.55	4.75	5.18	4.75	5.55
JDuaWild1	0.31*	0.18*	0.08*	0.06*	0.09*	0.04	0.10*	0.15*	0.11*	0.11*	0.09*	0.06	0.11*	0.11*	0.15*	0.13*	0.06	0.02	0.22	0.05	0.05	0.12	2.89	0.19	1.35	2.31	8.27	2.31	1.35
JDuaWild2	0.34*	0.18*	0.00	0.08*	0.08	0.03	0.09	0.20*	0.08	0.10*	0.05	0.12*	0.12*	0.12*	0.27*	0.15*	0.21*	0.06	0.05	0.13*	0.04	0.09	2.94	0.21	1.39	2.36	8.25	2.36	1.39
JDuaWild3	0.29*	0.10	-0.05	0.05	-0.02	-0.03	0.02	0.07	-0.05	0.05	-0.02	0.07	0.08	0.10	0.17	0.08	0.11	-0.01	0.03	-0.03	0.11*	0.07	2.91	0.17	1.36	2.35	8.29	2.35	1.36
JDuaWild4	0.28*	0.24*	-0.02	0.06	0.04	0.00	0.10	0.23*	0.10	0.15*	0.06	0.12	0.11*	0.08	0.24*	0.08	0.24*	0.06	0.05	-0.03	-0.02	0.35	2.94	0.16	1.37	2.40	8.32	2.40	1.37
CLopWild	0.06	0.20*	0.10*	0.10*	0.01	0.08	0.04	0.07	0.09*	0.09*	0.03	0.11	0.12*	0.09	0.07	0.11	0.18*	0.10	0.13*	0.14*	0.04	0.09	0.12*	2.78	1.68	1.07	10.33	1.07	1.68
JDuaWild5	0.20*	0.16*	0.01	0.05	0.01	0.01	0.07	0.12*	0.04	0.07	0.02	0.09	0.10*	0.06	0.18*	0.05	0.14*	0.01	0.04	0.00	-0.05	-0.02	0.03	0.43	1.20	2.27	8.46	2.27	1.20
LatR25	0.20*	0.10*	0.01	0.04	-0.01	0.07	0.04	0.08	0.03	0.09*	0.04	0.02	0.04	0.03	0.15*	0.07	0.10	0.02	0.04	0.04	-0.02	0.02	0.02	-0.01	0.46	1.55	9.46	1.55	0.00
LatR70	0.20*	0.17*	0.03	0.13*	0.01	0.09	0.07	0.09*	0.04	0.14*	0.05	0.10	0.11*	0.10	0.16*	0.02	0.15*	0.02	0.12*	0.09	-0.04	0.05	0.02	0.00	0.00	0.30	9.28	0.00	1.55
CLopR26	0.31*	0.35*	0.12*	0.16*	0.14*	0.24*	0.22*	0.32*	0.22*	0.32*	0.19*	0.21*	0.18*	0.12*	0.33*	0.06	0.35*	0.13*	0.18*	0.20*	0.20*	0.13*	0.15*	0.12*	0.07	0.09*	-0.09*	9.28	9.46
QuixR27	0.17*	0.22*	0.02	0.09*	0.00	0.08	0.04	0.15*	0.08	0.13*	0.05	0.08	0.06	0.04	0.17*	0.02	0.21*	0.04	0.11*	0.06	-0.02	0.00	0.02	0.00	0.00	-0.02	0.07	0.39	1.55
CLopR69	0.22*	0.17*	0.11*	0.10*	0.12*	0.11*	0.14*	0.17*	0.11*	0.13*	0.11*	0.16*	0.17*	0.13	0.27*	0.25*	0.22*	0.18*	0.15*	0.12*	0.09	0.10	0.12*	0.08	0.05	0.15*	0.25*	0.13*	0.48

*p ≤ 0.05. Samples (locality name, domiciliary unit identification). R: Remot; Wild: wild ecotope; Lat: Latadas; Clop: Cipriano Lopes; Quix: Quixabinha; Jen: Jenipapeiro; JDua: João Duarte.

## DISCUSSION

Previous studies conducted in the North-East region of Brazil have reported that *T. brasiliensis* is the most prevalent triatomine in domestic environments.^55^ ^,^ ^56^ This species can form large colonies and has high levels of natural *T. cruzi* infection.^56^

In our study, the number of alleles per locus (two to 14) is lower than those observed by other authors studying the same species in north-east Brazil.^18^ ^,^ ^20^ The population with the highest average number of alleles per locus (4.2) was of sylvatic origin (João Duarte locality, JDuaWild1), corroborating previous studies in the State of Ceará.^18^ Almeida et al.^20^ observed that the sylvatic populations they studied had higher average NA compared to peridomiciliary populations.

In our study, the *T. brasiliensis* population from the Latadas locality (peridomicile, DU18c1) exhibited the lowest average NA and AR, along with the highest *F*is value (0.44), suggesting a heterozygosity deficit, the presence of null alleles, or population substructure. The heterozygosity deficit may result from persistent infestations by individuals that survived insecticide spraying, leading to increased inbreeding,^57^ and/or from mating among individuals restricted to certain sylvatic habitats. The fixation indices further support this, as they showed significant values (p ≤ 0.05), indicating inbreeding, which may reflect both mating among related individuals and the presence of subpopulation structure.

In Jaguaruana, the most frequent sylvatic ecotope of *T. brasiliensis* is the cactus *Pilosocereus gounellei*, which is distributed in discontinuous clumps. This ecological context is contrary to that observed in Tauá, which differs from regions where triatomines are associated with granite outcrops (Fig. 1) and there is evidence of wide dispersal without cluster formation, characterising panmictic populations.^18^

The distribution pattern of triatomines in sylvatic environments certainly influences the reinfestation process in the anthropic and/or disturbed natural environments (*i.e.*, the intra- and peridomiciles). In Tauá, the area studied by Bezerra et al.,^18^ the population density of triatomines within DUs recovers completely one year after spraying with residual insecticide. In contrast, in the municipality of Tamboril, also in the State of Ceará, and with a landscape similar to that of Jaguaruana, triatomine infestation remained low compared to original data over the same period.^58^ These findings reinforce the ability and sensitivity of microsatellite markers for investigating the population dynamics of triatomines.

The NJ dendrogram suggests that the two peridomiciliary annexes of Cristiano Lopes’ DU 23 (CLop23p1 and CLop23p2) had the same source of infestation or that one colonisation gave rise to another. A different situation occurred in the peridomiciliary annexes of DU 33 in the same locality (CLop33p1 and CLop33p2), where the sources of infestation probably were different. Although the branches did not show statistical support, the dendrogram suggests that the sylvatic focus found in Cristiano Lopes may have been responsible for the invasion of *T. brasiliensis* in DUs 17, 27, and 15c2 of the same locality (CLop17, CLop27, and CLop15c2).

The Bayesian STRUCTURE analysis corroborated the dendrogram observations, both approaches provided complementary insights into population differentiation and gene flow, revealing multiple genetic clusters and varying degrees of admixture among the triatomine populations that we studied. The Lat18C1 population exhibited a homogeneous genetic composition, consistent with the differentiation observed in the NJ tree. The JDuaWild and Lat populations shared significant proportions of genetic ancestry, supporting their genetic proximity as inferred from the NJ analysis. In contrast, the CLop and Jen populations displayed a high degree of genetic admixture, suggesting a history of gene flow between these lineages. Additionally, some CLop populations (CLop23p1 and CLop23p2) exhibited similar genetic profiles, indicating a close and possibly recent relationship (Fig. 4).

Susceptibility tests to pyrethroid insecticides show that the samples analysed here are susceptible to these insecticides (unpublished results obtained by REMOT), indicating that the persistence of triatomine infestation is not due to insecticide resistance. Our results emphasise the complexity of *T. brasiliensis* control, and highlight the difficulties and possible operational shortcomings, especially in the peridomiciliary environment. This fact is understandable given the numerous hiding places that are inaccessible to insecticide spraying both within the home and in their peridomiciliary annexes, even considering the residual activity of the insecticide indoors.^13^ ^,^ ^59^ ^,^ ^60^

In conclusion, our study provides key insights into the genetic structure and population dynamics of *T. brasiliensis* in Jaguaruana. The observed complexity, with anthropogenic environments colonised from diverse sources, emphasises the challenges faced in vector control. This necessitates tailored strategies that consider regional variations and the adaptability of *T. brasiliensis*. Effective surveillance and control planning must address not only existing infestations but also the intricate processes influencing vector dynamics. By enhancing our understanding of these complexities, we pave the way for more targeted and sustainable CD control efforts in our study region.

## SUPPLEMENTARY MATERIALS

Supplementary material

## Data Availability

The datasets used and/or analysed during the current study are available from the corresponding author on reasonable request.
