# Supplementary material for: The complexity of the population dynamics of Triatoma brasiliensis in rural north-east Brazil indicated by genetic characterisation
Source: Mem Inst Oswaldo Cruz. 2026 Mar 30;121:e250076. doi: 10.1590/0074-02760250076 (PMC13035281; doi:10.1590/0074-02760250076)
Supplement: Supplementary material [file 1678-8060-mioc-121-e250076-s1.pdf]

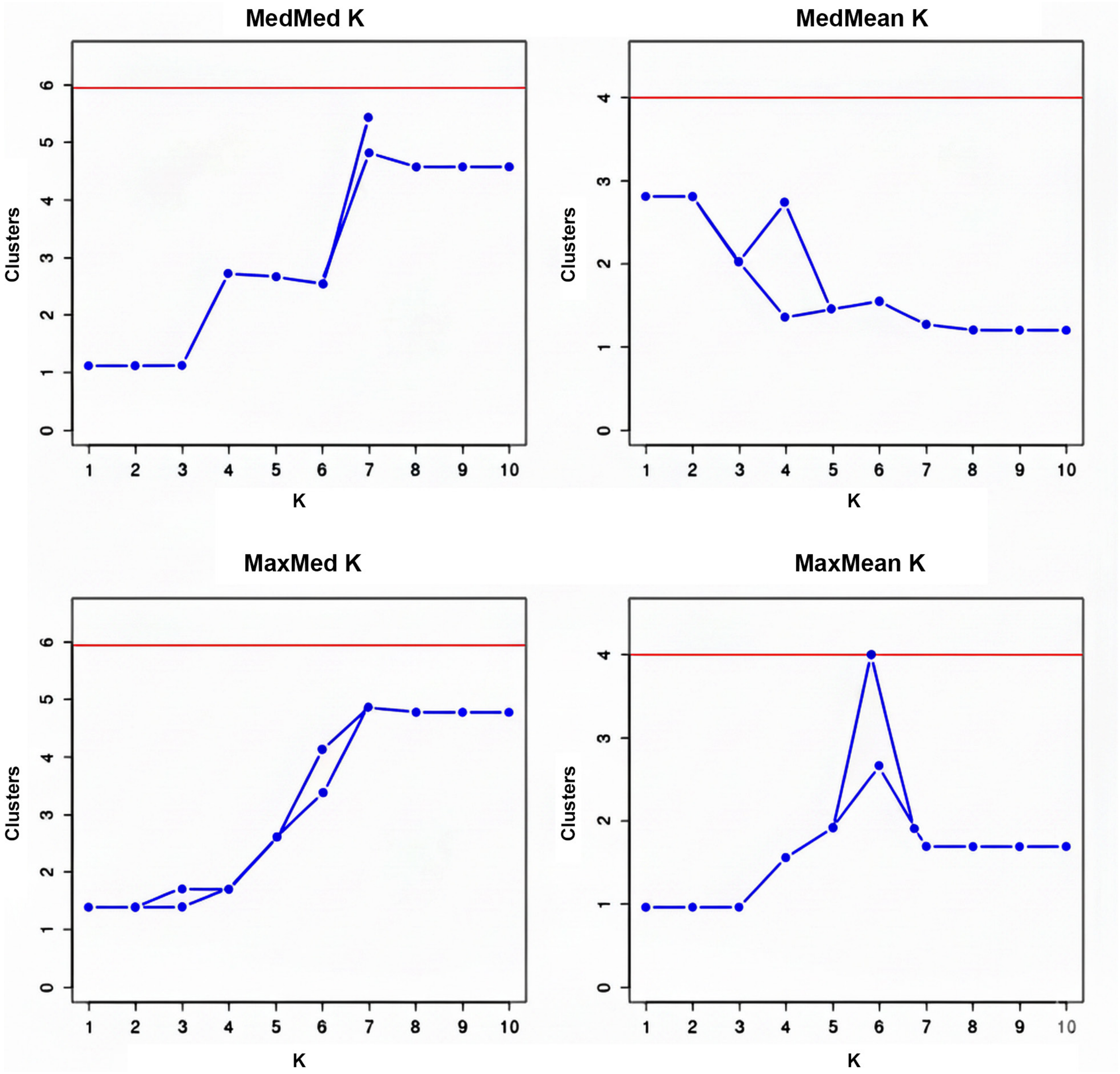

The best K value identified by the metrics MedMedK, MedMeanK, MaxMedK, and MaxMeanK ranging from 1 to 10.

TABLE I

Alleles size per locus and sample of *Triatoma brasiliensis* from Jaguaruana, Ceará, Brasil

Sample	Tb728	Tb830	Tb860	Tb7180	Tb8124	Total
Lat18c1	308; 310	284	394; 396	236; 238; 246	234; 236	10
Lat23	310; 312	276; 282; 284	394	228 ;230; 236; 240; 244	232; 236	13
Lat3c1	308; 310	276; 282; 284	394; 396	234; 236; 238; 240; 244	232; 234; 236; 240; 242	17
Lat11	308; 310; 312; 316	276; 282; 284	394; 396	216; 236; 238; 244	228; 230; 232; 236; 238	18
Lat13	308; 310; 312	276; 282; 284; 290	394; 396	228 ;230; 236; 238; 244	230; 232; 234; 236	18
Lat14c1	308; 310; 312; 316	276; 282; 284	394; 396	224; 236; 238; 240; 244	224; 230; 232; 234; 236	19
CLop17	308; 310; 312	276; 284; 290	394; 396	216; 224; 234; 236	224; 230; 232; 238	16
CLop33c1p1	310; 312	282; 284	394; 396	234; 236; 238; 242; 244	232; 234	13
CLop33c1p2	308; 310; 312; 316	276; 282; 284	394; 396	216; 236; 238; 242; 244	224; 230; 232; 234	19
CLop15c2	308; 310; 312	276; 284	394; 396	216; 230; 232; 236; 238; 240	232; 236	15
CLop27	308; 310; 312	276; 282; 284; 290	394; 396	216; 228; 236; 238; 244; 246	230; 232; 234; 236	19
CLop23p1	308; 310	276; 282; 284; 290	394; 396	216; 224; 228; 234; 236; 244	232; 234; 236	17
CLop23p2	308; 310	276; 282; 284; 290	394	216; 228; 234; 236; 238; 244	230; 232; 234; 236	17
Quix5	308; 310	276; 282; 284	394	216; 236; 244	224; 228; 234; 242	14
Jen6	308; 310	282; 284	394; 396	234; 236; 238	230; 232; 234	12
Jen1	310; 312	276; 282; 284	394; 396	216; 230; 232; 236; 238	232; 234	14
Jen6c1	310; 312	282; 284	394; 396	234; 236; 238; 244	232	11
Jen15	308; 310; 312	276; 282; 284	394; 396	216; 234; 236; 238; 240; 244	226; 232; 234	17
JDuaWild1	308; 310; 312; 316	276; 282; 284	394; 396	228; 234; 236; 238; 240; 244	230; 232; 234; 236; 238	21
JDuaWild2	308; 310; 312; 316	276; 284	394	216; 228; 234; 236; 238; 240; 244	232; 234; 236	17
JDuaWild3	308; 310; 312; 316	276; 284	394; 396	216; 236; 238; 240; 244	232; 234	15
JDuaWild4	308; 310; 316	276; 284	394; 396	228; 234; 236; 238; 240	230; 232; 234; 236	17
CLopWild	308; 310; 312	276; 284	394; 396	226; 228; 236; 238; 244	232; 234; 236	15
JDuaWild5	308; 310; 312; 316	276; 282; 284	394	216; 230; 236; 238; 240; 242; 244	232; 234; 236	19
LatR25	310; 312; 316	276; 282; 284	394; 396	228; 234; 236; 238; 240; 244	232; 234; 236	17
LatR70	308; 310; 312	276; 282; 284	394; 396	216; 234; 236; 238; 244	232; 234	15
CLopR26	310; 312	276; 284	394; 396	214; 216; 236; 244	230; 232; 234; 236	14
QuixR27	308; 310; 312	276; 284	394; 396	216; 228; 234; 236; 238; 244	230; 232; 234; 236	17
CLopR69	308; 310; 312	276; 282; 284	394	234; 236; 238; 240; 242; 244	232; 234; 236; 238	17
Alelleles per locus	4	4	2	14	10	-

Samples (locality name, domiciliary unit identification). R: Remot; Wild: wild ecotope; Lat: Latadas; CLop: Cipriano Lopes; Quix: Quixabinha; Jen: Jenipapeiro; JDua: João Duarte.

TABLE II

Pairwise *Fst* above of diagonal, and correct pairwise *Fst* without null alleles

		1	2	3	4	5	6	7	8	9	10	11	12	13	14	15	16	17	18	19	20	21	22	23	24	25	26	27	28	29
Lat18c1	1		0,38	0,28	0,22	0,18	0,20	0,20	0,24	0,27	0,23	0,16	0,26	0,29	0,23	0,21	0,28	0,40	0,28	0,28	0,30	0,23	0,23	0,02	0,21	0,17	0,17	0,32	0,15	0,20
Lat23	**2**	0,45		0,12	0,16	0,11	0,18	0,13	0,08	0,01	0,15	0,14	0,14	0,15	0,23	0,31	0,29	0,13	0,17	0,17	0,16	0,08	0,20	0,19	0,13	0,08	0,14	0,28	0,17	0,12
Lat3c1	**3**	0,30	0,13		0,07	0,02	0,05	0,07	0,14	0,03	0,13	0,08	0,08	0,07	0,08	0,22	0,10	0,19	0,03	0,08	0,00	0,00	0,00	0,11	0,01	0,01	0,03	0,11	0,01	0,07
Lat11	**4**	0,26	0,18	0,08		0,06	0,05	0,06	0,14	0,09	0,07	0,06	0,10	0,09	0,07	0,17	0,17	0,15	0,07	0,05	0,06	0,04	0,05	0,08	0,04	0,02	0,10	0,14	0,06	0,08
Lat13	**5**	0,20	0,13	0,01	0,06		0,08	0,00	0,06	0,03	0,08	0,04	0,01	0,01	0,06	0,11	0,06	0,13	0,02	0,08	0,06	-0,01	0,02	0,02	0,03	0,00	0,00	0,12	0,00	0,08
Lat14c1	**6**	0,23	0,18	0,05	0,05	0,07		0,06	0,12	0,06	0,09	0,02	0,13	0,16	0,14	0,14	0,14	0,15	0,07	0,03	0,02	0,00	0,00	0,07	0,00	0,05	0,07	0,22	0,05	0,08
CLop17	**7**	0,25	0,16	0,08	0,06	0,00	0,07		0,02	0,04	0,04	0,01	0,04	0,06	0,11	0,09	0,13	0,12	0,06	0,09	0,07	0,01	0,06	0,03	0,06	0,02	0,03	0,21	0,02	0,11
CLop33c1p1	**8**	0,30	0,09	0,16	0,17	0,08	0,13	0,04		0,03	0,08	0,07	0,10	0,15	0,22	0,11	0,19	0,03	0,10	0,13	0,17	0,06	0,18	0,07	0,12	0,05	0,06	0,28	0,10	0,13
CLop33c1p2	**9**	0,31	0,01	0,03	0,09	0,03	0,05	0,05	0,03		0,09	0,05	0,09	0,10	0,13	0,20	0,16	0,10	0,08	0,10	0,07	0,00	0,08	0,10	0,04	0,02	0,02	0,20	0,06	0,07
CLop15c2	**10**	0,28	0,15	0,12	0,08	0,05	0,08	0,04	0,08	0,08		0,04	0,14	0,14	0,20	0,19	0,25	0,14	0,12	0,12	0,09	0,03	0,13	0,09	0,06	0,09	0,13	0,30	0,11	0,10
CLop27	**11**	0,19	0,14	0,07	0,08	0,04	0,02	0,01	0,07	0,05	0,04		0,08	0,11	0,11	0,11	0,14	0,12	0,08	0,08	0,05	0,00	0,04	0,04	0,02	0,04	0,05	0,20	0,03	0,07
CLop23p1	**12**	0,30	0,14	0,07	0,12	0,01	0,13	0,03	0,11	0,09	0,12	0,08		-0,03	0,08	0,10	0,15	0,11	0,09	0,08	0,10	0,08	0,09	0,11	0,10	0,03	0,08	0,18	0,06	0,12
CLop23p2	**13**	0,31	0,17	0,07	0,09	0,01	0,16	0,04	0,17	0,11	0,12	0,10	0,00		0,05	0,18	0,17	0,18	0,10	0,12	0,10	0,09	0,09	0,13	0,10	0,04	0,10	0,15	0,06	0,12
Quix5	14	0,26	0,28	0,08	0,07	0,07	0,13	0,10	0,26	0,14	0,21	0,12	0,10	0,07		0,22	0,16	0,28	0,12	0,13	0,11	0,09	0,07	0,10	0,09	0,04	0,09	0,09	0,05	0,12
Jen6	**15**	0,24	0,30	0,22	0,19	0,10	0,13	0,06	0,08	0,19	0,16	0,11	0,09	0,17	0,24		0,21	0,14	0,16	0,14	0,25	0,18	0,20	0,07	0,20	0,13	0,14	0,31	0,14	0,25
Jen1	**16**	0,30	0,32	0,10	0,19	0,06	0,15	0,15	0,23	0,17	0,26	0,13	0,16	0,17	0,18	0,23		0,23	0,03	0,13	0,15	0,09	0,08	0,13	0,09	0,07	0,02	0,10	0,02	0,22
Jen6c1	**17**	0,43	0,12	0,18	0,16	0,10	0,12	0,11	0,02	0,09	0,13	0,11	0,07	0,16	0,29	0,09	0,24		0,07	0,07	0,20	0,11	0,22	0,20	0,15	0,10	0,14	0,33	0,18	0,21
Jen15	**18**	0,32	0,18	0,03	0,08	0,01	0,07	0,07	0,12	0,07	0,11	0,07	0,08	0,09	0,14	0,16	0,03	0,07		0,02	0,05	0,00	0,03	0,10	0,02	0,01	0,02	0,11	0,02	0,14
JDuaWild1	**19**	0,31	0,17	0,08	0,05	0,07	0,03	0,08	0,14	0,10	0,11	0,08	0,06	0,10	0,14	0,13	0,13	0,04	0,01		0,04	0,05	0,04	0,13	0,04	0,04	0,11	0,18	0,09	0,12
JDuaWild2	**20**	0,33	0,17	0,00	0,07	0,05	0,01	0,07	0,19	0,06	0,09	0,04	0,10	0,10	0,12	0,25	0,15	0,20	0,05	0,04		0,00	0,00	0,14	0,00	0,04	0,08	0,18	0,03	0,08
JDuaWild3	**21**	0,29	0,08	0,00	0,07	0,00	0,00	0,02	0,06	0,00	0,03	0,00	0,06	0,08	0,12	0,16	0,09	0,09	0,00	0,03	0,00		0,00	0,05	0,00	0,00	0,00	0,17	0,00	0,05
JDuaWild4	**22**	0,25	0,23	0,00	0,06	0,02	0,00	0,08	0,21	0,08	0,14	0,05	0,10	0,10	0,07	0,21	0,07	0,21	0,04	0,04	0,00	0,00		0,08	0,00	0,00	0,04	0,13	0,00	0,07
CLopWild	**23**	0,05	0,20	0,09	0,09	0,00	0,06	0,02	0,06	0,07	0,08	0,02	0,09	0,11	0,11	0,05	0,11	0,17	0,09	0,12	0,12	0,04	0,07		0,07	0,02	0,02	0,17	0,02	0,10
JDuaWild5	**24**	0,20	0,13	0,00	0,04	0,00	0,00	0,06	0,11	0,02	0,05	0,01	0,07	0,08	0,06	0,17	0,06	0,12	0,00	0,03	0,00	0,00	0,00	0,03		0,00	0,03	0,14	0,00	0,04
LatR25	**25**	0,19	0,08	0,00	0,02	0,00	0,05	0,02	0,06	0,01	0,07	0,03	0,00	0,01	0,03	0,13	0,06	0,07	0,00	0,03	0,02	0,00	0,00	0,00	0,00		0,00	0,07	0,00	0,03
LatR70	**26**	0,20	0,16	0,02	0,12	0,00	0,07	0,05	0,08	0,03	0,13	0,04	0,09	0,10	0,11	0,14	0,01	0,13	0,01	0,10	0,08	0,00	0,03	0,01	0,00	0,00		0,10	0,00	0,10
CLopR26	**27**	0,33	0,34	0,12	0,16	0,14	0,24	0,23	0,33	0,22	0,32	0,21	0,20	0,17	0,11	0,34	0,10	0,36	0,14	0,19	0,18	0,20	0,13	0,17	0,11	0,06	0,11		0,08	0,21
QuixR27	**28**	0,16	0,20	0,01	0,08	0,00	0,06	0,02	0,13	0,07	0,12	0,03	0,06	0,05	0,05	0,15	0,01	0,18	0,02	0,10	0,03	0,00	0,00	0,00	0,00	0,00	0,00	0,07		0,08
CLopR69	**29**	0,22	0,15	0,10	0,09	0,10	0,09	0,12	0,16	0,09	0,11	0,09	0,13	0,15	0,12	0,25	0,24	0,19	0,17	0,14	0,10	0,07	0,07	0,10	0,05	0,02	0,13	0,23	0,11	

Samples (locality name, domiciliary unit identification). R: Remot; Wild: wild ecotope; Lat: Latadas; Clop: Cipriano Lopes; Quix: Quixabinha; Jen: Jenipapeiro; JDua: João Duarte.

TABLE III

Maximum values (below the diagonal) and minimum values (above the diagonal) of the 95% confidence interval for pairwise *Fst* analysis using null alleles

		1	2	3	4	5	6	7	8	9	10	11	12	13	14	15	16	17	18	19	20	21	22	23	24	25	26	27	28	29
Lat18c1	1		0,18	0,12	0,13	0,02	0,10	0,01	0,03	0,05	0,09	0,08	0,04	0,10	0,09	0,00	0,06	0,12	0,13	0,15	0,23	0,08	0,06	0,00	0,13	0,08	0,05	0,00	0,00	0,12
Lat23	**2**	0,59		0,10	0,07	0,03	0,05	0,02	0,00	0,00	0,00	0,06	0,05	0,08	0,02	0,00	0,17	0,04	0,12	0,09	0,12	0,00	0,15	0,07	0,03	0,00	0,05	0,06	0,08	0,00
Lat3c1	**3**	0,46	0,16		0,03	0,00	0,00	0,01	0,07	0,00	0,04	0,00	0,00	0,00	0,00	0,05	0,03	0,04	0,00	0,00	0,00	0,00	0,00	0,00	0,00	0,00	0,00	0,05	0,00	0,00
Lat11	**4**	0,35	0,27	0,11		0,00	0,01	0,03	0,06	0,01	0,00	0,05	0,05	0,01	0,00	0,10	0,08	0,01	0,03	0,02	0,00	0,00	0,00	0,04	0,00	0,00	0,03	0,04	0,01	0,05
Lat13	**5**	0,31	0,22	0,05	0,12		0,00	0,00	0,03	0,00	0,02	0,00	0,00	0,00	0,00	0,00	0,02	0,00	0,00	0,00	0,00	0,00	0,00	0,00	0,00	0,00	0,00	0,04	-0,04	0,04
Lat14c1	**6**	0,28	0,27	0,14	0,10	0,21		0,00	0,03	0,00	0,03	0,00	0,01	0,01	0,06	0,02	0,08	0,02	0,00	0,00	0,00	0,00	0,00	0,00	0,00	0,00	0,00	0,13	0,00	0,00
CLop17	**7**	0,41	0,27	0,13	0,10	0,03	0,15		0,00	0,00	0,00	0,00	0,00	0,00	0,00	0,00	0,06	0,01	0,00	0,00	0,03	0,00	0,04	0,00	0,01	0,00	0,00	0,05	0,00	0,02
CLop33c1p1	**8**	0,53	0,21	0,30	0,27	0,14	0,22	0,08		0,00	0,04	0,03	0,01	0,06	0,05	0,00	0,04	0,00	0,01	0,03	0,11	0,00	0,12	0,00	0,05	0,00	0,00	0,01	0,02	0,00
CLop33c1p2	**9**	0,42	0,05	0,08	0,14	0,11	0,14	0,15	0,06		0,00	0,00	0,00	-0,01	0,00	0,00	0,08	0,03	0,01	0,01	0,02	0,00	0,03	0,00	0,00	0,00	0,00	0,02	0,00	0,00
CLop15c2	**10**	0,48	0,26	0,21	0,17	0,10	0,10	0,10	0,14	0,16		0,00	0,00	0,00	0,00	0,04	0,11	0,03	0,03	0,02	0,03	0,00	0,05	0,00	0,00	0,02	0,07	0,08	0,03	0,00
CLop27	**11**	0,26	0,19	0,17	0,10	0,10	0,07	0,04	0,10	0,09	0,08		0,00	0,00	0,04	0,00	0,07	0,04	0,00	0,00	0,00	0,00	0,00	0,00	0,00	0,00	0,00	0,08	0,00	0,03
CLop23p1	**12**	0,47	0,22	0,15	0,17	0,08	0,23	0,13	0,23	0,16	0,28	0,17		0,00	0,00	0,00	0,12	0,00	0,00	0,00	0,00	0,00	0,04	0,00	0,01	0,00	0,02	0,05	0,00	0,04
CLop23p2	**13**	0,47	0,27	0,14	0,21	0,07	0,33	0,14	0,29	0,18	0,27	0,23	0,00		0,00	0,00	0,07	0,05	0,00	0,00	0,00	0,00	0,02	0,00	0,01	0,00	0,04	0,10	0,01	0,08
Quix5	**14**	0,45	0,49	0,14	0,16	0,19	0,19	0,24	0,46	0,25	0,40	0,19	0,23	0,23		0,10	0,04	0,08	0,01	0,01	0,00	0,00	0,00	0,02	0,00	0,00	0,00	0,02	0,00	0,01
Jen6	15	0,40	0,50	0,35	0,33	0,17	0,23	0,12	0,15	0,34	0,33	0,22	0,22	0,40	0,36		0,08	-0,04	0,03	0,03	0,07	0,00	0,08	0,01	0,06	0,05	0,05	0,21	0,07	0,07
Jen1	**16**	0,54	0,47	0,21	0,30	0,11	0,22	0,23	0,39	0,23	0,42	0,22	0,21	0,36	0,32	0,32		0,07	0,00	0,08	0,04	0,03	0,00	0,00	0,00	0,00	0,00	0,00	0,00	0,09
Jen6c1	**17**	0,63	0,22	0,29	0,29	0,20	0,21	0,20	0,12	0,14	0,29	0,17	0,20	0,34	0,49	0,20	0,44		0,00	0,00	0,11	0,01	0,08	0,02	0,00	0,00	0,00	0,03	0,01	0,00
Jen15	**18**	0,49	0,23	0,12	0,14	0,10	0,16	0,12	0,28	0,12	0,23	0,17	0,18	0,25	0,25	0,24	0,08	0,23		0,00	0,00	0,00	0,00	0,00	0,00	0,00	0,00	0,03	0,00	0,04
JDuaWild1	**19**	0,43	0,23	0,17	0,08	0,21	0,10	0,17	0,24	0,16	0,22	0,16	0,23	0,31	0,24	0,20	0,16	0,08	0,04		0,00	0,00	0,01	0,00	0,00	0,00	0,03	0,10	0,02	0,07
JDuaWild2	**20**	0,44	0,21	0,04	0,14	0,16	0,07	0,15	0,29	0,10	0,16	0,14	0,20	0,26	0,20	0,41	0,32	0,29	0,12	0,09		0,00	0,00	0,00	0,00	0,00	0,00	0,14	0,00	0,04
JDuaWild3	**21**	0,42	0,15	0,02	0,14	0,02	0,01	0,09	0,09	0,03	0,08	0,04	0,15	0,15	0,25	0,31	0,17	0,19	0,05	0,09	0,01		0,00	0,00	0,00	0,00	0,00	0,07	0,00	0,00
JDuaWild4	**22**	0,46	0,30	-0,01	0,15	0,10	0,04	0,10	0,31	0,11	0,22	0,12	0,15	0,21	0,14	0,32	0,17	0,33	0,11	0,07	0,01	0,01		0,00	0,00	0,00	0,00	0,01	0,00	0,03
CLopWild	**23**	0,12	0,30	0,20	0,16	0,04	0,15	0,07	0,15	0,16	0,18	0,07	0,23	0,27	0,19	0,11	0,25	0,30	0,20	0,22	0,24	0,12	0,16		0,00	0,00	0,00	0,03	0,00	0,01
JDuaWild5	**24**	0,28	0,23	0,03	0,11	0,05	0,03	0,11	0,21	0,07	0,15	0,06	0,14	0,16	0,15	0,31	0,22	0,26	0,06	0,07	0,00	0,05	0,03	0,13		0,00	0,00	0,05	0,00	0,00
LatR25	**25**	0,31	0,16	0,04	0,07	0,00	0,15	0,05	0,17	0,04	0,11	0,08	0,05	0,07	0,12	0,23	0,15	0,19	0,03	0,11	0,09	0,01	0,07	0,07	0,01		0,00	0,00	0,00	0,00
LatR70	**26**	0,30	0,27	0,08	0,18	0,01	0,19	0,11	0,17	0,07	0,21	0,09	0,14	0,19	0,21	0,24	0,05	0,26	0,08	0,19	0,18	0,00	0,10	0,08	0,09	0,02		0,07	0,00	0,21
CLopR26	**27**	0,65	0,52	0,17	0,25	0,20	0,35	0,35	0,55	0,36	0,51	0,36	0,30	0,24	0,20	0,46	0,20	0,56	0,23	0,25	0,21	0,30	0,21	0,37	0,20	0,14	0,18		0,01	0,24
QuixR27	**28**	0,32	0,30	0,02	0,13	-0,01	0,15	0,10	0,25	0,11	0,22	0,08	0,15	0,11	0,13	0,20	0,06	0,32	0,11	0,19	0,11	0,00	0,03	0,06	0,04	0,00	0,02	0,14		0,16
CLopR69	**29**	0,26	0,36	0,20	0,12	0,18	0,18	0,24	0,40	0,25	0,25	0,17	0,21	0,20	0,23	0,42	0,38	0,43	0,28	0,19	0,17	0,23	0,15	0,21	0,15	0,08	0,26	0,41	0,21	

Samples (locality name, domiciliary unit identification). R: Remot; Wild: wild ecotope; Lat: Latadas; Clop: Cipriano Lopes; Quix: Quixabinha; Jen: Jenipapeiro; JDua: João Duarte

TABLE IV

Maximum values (below the diagonal) and minimum values (above the diagonal) of the 95% confidence interval for pairwise *Fst* analysis without null alleles

		1	2	3	4	5	6	7	8	9	10	11	12	13	14	15	16	17	18	19	20	21	22	23	24	25	26	27	28	29
Lat18c1	1		0,20	0,09	0,11	0,02	0,08	0,01	0,05	0,08	0,08	0,06	0,02	0,09	0,07	0,02	0,03	0,10	0,09	0,11	0,20	0,08	0,06	0,00	0,00	0,06	0,01	0,00	0,00	0,12
Lat23	**2**	0,48		0,09	0,06	0,02	0,06	0,01	0,00	0,00	0,00	0,06	0,06	0,07	0,01	0,01	0,16	0,07	0,10	0,08	0,09	0,00	0,13	0,08	0,05	0,00	0,04	0,06	0,06	0,00
Lat3c1	**3**	0,45	0,14		0,02	0,00	0,00	0,02	0,06	0,00	0,03	0,00	0,00	0,00	0,00	0,04	0,03	0,06	0,00	0,00	0,00	0,00	0,00	0,00	0,00	0,00	0,00	0,05	0,00	0,00
Lat11	**4**	0,33	0,22	0,12		0,01	0,01	0,03	0,08	0,03	0,00	0,04	0,03	0,01	0,00	0,08	0,08	0,01	0,03	0,02	0,00	0,00	0,00	0,02	0,00	0,00	0,03	0,04	0,01	0,04
Lat13	**5**	0,33	0,20	0,05	0,12		0,00	0,00	0,03	0,00	0,03	0,00	0,00	0,00	0,00	0,02	0,02	0,02	0,00	0,00	0,00	0,00	0,00	0,00	0,00	0,00	0,00	0,03	0,00	0,03
Lat14c1	**6**	0,27	0,27	0,13	0,10	0,22		0,00	0,02	0,00	0,05	0,00	0,02	0,03	0,07	0,04	0,07	0,03	0,00	0,00	0,00	0,00	0,00	0,00	0,00	0,00	0,00	0,10	0,00	0,00
CLop17	**7**	0,34	0,24	0,11	0,10	0,01	0,15		0,00	0,00	0,01	0,00	0,00	0,00	0,00	0,00	0,06	0,02	0,00	0,00	0,03	0,00	0,03	0,00	0,01	0,00	0,00	0,05	0,00	0,01
CLop33c1p1	**8**	0,43	0,20	0,25	0,21	0,10	0,22	0,04		0,00	0,03	0,01	0,03	0,07	0,07	0,02	0,04	0,00	0,01	0,04	0,10	0,01	0,10	0,00	0,07	0,00	0,00	0,02	0,02	0,00
CLop33c1p2	**9**	0,37	0,05	0,10	0,14	0,09	0,15	0,12	0,05		0,03	0,00	0,00	0,02	0,00	0,00	0,08	0,05	0,02	0,02	0,03	0,00	0,04	0,01	0,01	0,00	0,00	0,02	0,00	0,00
CLop15c2	**10**	0,40	0,26	0,21	0,14	0,13	0,12	0,09	0,15	0,16		0,01	0,00	0,00	0,02	0,06	0,11	0,04	0,03	0,04	0,04	0,00	0,04	0,01	0,00	0,04	0,08	0,08	0,03	0,00
CLop27	**11**	0,21	0,19	0,16	0,09	0,12	0,06	0,05	0,11	0,10	0,08		0,00	0,00	0,04	0,00	0,08	0,04	0,00	0,00	0,00	0,00	0,00	0,00	0,00	0,00	0,00	0,08	0,00	0,03
CLop23p1	**12**	0,49	0,21	0,16	0,17	0,06	0,24	0,14	0,19	0,15	0,30	0,17		0,00	0,00	0,00	0,11	0,00	0,00	0,00	0,01	0,00	0,02	0,00	0,02	0,00	0,02	0,05	0,00	0,06
CLop23p2	**13**	0,46	0,25	0,13	0,20	0,07	0,33	0,14	0,24	0,15	0,27	0,23	0,00		0,00	0,02	0,07	0,06	0,01	0,00	0,00	0,00	0,02	0,00	0,01	0,00	0,04	0,10	0,01	0,08
Quix5	**14**	0,45	0,40	0,14	0,16	0,16	0,20	0,22	0,37	0,22	0,36	0,16	0,18	0,19		0,08	0,03	0,07	0,01	0,01	0,00	0,00	0,00	0,01	0,00	0,00	0,00	0,02	0,00	0,02
Jen6	**15**	0,38	0,50	0,35	0,33	0,19	0,24	0,18	0,20	0,36	0,34	0,22	0,22	0,40	0,35		0,06	0,02	0,05	0,04	0,06	0,00	0,07	0,01	0,07	0,05	0,04	0,19	0,06	0,06
Jen1	16	0,53	0,43	0,21	0,28	0,11	0,21	0,19	0,32	0,22	0,39	0,21	0,21	0,36	0,30	0,31		0,07	0,00	0,08	0,04	0,03	0,00	0,00	0,00	0,00	0,00	0,00	0,00	0,08
Jen6c1	**17**	0,62	0,23	0,28	0,28	0,24	0,25	0,19	0,12	0,15	0,29	0,21	0,27	0,38	0,49	0,24	0,45		0,00	0,00	0,13	0,02	0,08	0,02	0,04	0,00	0,00	0,03	0,02	0,03
Jen15	**18**	0,47	0,22	0,12	0,11	0,11	0,16	0,12	0,22	0,12	0,23	0,16	0,18	0,26	0,22	0,23	0,06	0,20		0,00	0,00	0,00	0,00	0,00	0,00	0,00	0,00	0,03	0,00	0,05
JDuaWild1	**19**	0,43	0,23	0,16	0,07	0,21	0,10	0,18	0,20	0,16	0,22	0,15	0,23	0,32	0,23	0,21	0,17	0,16	0,04		0,00	0,00	0,00	0,01	0,01	0,00	0,03	0,09	0,02	0,06
JDuaWild2	**20**	0,43	0,21	0,03	0,14	0,16	0,09	0,15	0,25	0,10	0,16	0,12	0,19	0,25	0,18	0,41	0,33	0,30	0,13	0,08		0,00	0,00	0,05	0,00	0,00	0,00	0,14	0,00	0,05
JDuaWild3	**21**	0,35	0,15	0,02	0,11	0,03	0,02	0,07	0,08	0,01	0,09	0,04	0,16	0,15	0,17	0,32	0,17	0,22	0,06	0,09	0,01		0,00	0,00	0,00	0,00	0,00	0,06	0,00	0,00
JDuaWild4	**22**	0,45	0,25	0,01	0,13	0,10	0,04	0,10	0,26	0,10	0,21	0,11	0,14	0,21	0,14	0,31	0,17	0,39	0,10	0,07	0,01	0,01		0,01	0,00	0,00	0,00	0,02	0,00	0,03
CLopWild	**23**	0,10	0,29	0,20	0,16	0,08	0,15	0,08	0,16	0,17	0,18	0,08	0,25	0,27	0,19	0,14	0,25	0,36	0,21	0,22	0,24	0,12	0,15		0,00	0,00	0,00	0,03	0,00	0,03
JDuaWild5	**24**	0,30	0,18	0,05	0,10	0,12	0,04	0,11	0,18	0,07	0,12	0,06	0,17	0,23	0,15	0,34	0,24	0,29	0,07	0,07	0,01	0,04	0,02	0,15		0,00	0,00	0,08	0,00	0,00
LatR25	**25**	0,31	0,17	0,04	0,06	0,00	0,14	0,06	0,14	0,05	0,15	0,07	0,06	0,10	0,12	0,20	0,13	0,29	0,02	0,10	0,10	0,01	0,06	0,08	0,03		0,00	0,00	0,00	0,00
LatR70	**26**	0,28	0,24	0,08	0,16	0,02	0,19	0,09	0,14	0,06	0,19	0,10	0,13	0,19	0,17	0,26	0,05	0,30	0,07	0,19	0,19	0,01	0,10	0,09	0,12	0,02		0,13	0,00	0,00
CLopR26	**27**	0,64	0,43	0,15	0,21	0,17	0,33	0,31	0,48	0,33	0,47	0,35	0,29	0,19	0,15	0,42	0,20	0,55	0,17	0,23	0,20	0,27	0,20	0,39	0,21	0,15	0,18		0,00	0,04
QuixR27	**28**	0,32	0,25	0,03	0,10	0,01	0,13	0,09	0,20	0,10	0,19	0,07	0,14	0,12	0,11	0,20	0,07	0,35	0,08	0,16	0,10	0,00	0,02	0,07	0,06	0,00	0,01	0,14		0,01
CLopR69	**29**	0,26	0,30	0,16	0,10	0,14	0,16	0,21	0,32	0,20	0,22	0,14	0,17	0,15	0,21	0,42	0,38	0,45	0,23	0,16	0,13	0,16	0,12	0,21	0,10	0,09	0,22	0,38	0,16	

Samples (locality name, domiciliary unit identification). R: Remot; Wild: wild ecotope; Lat: Latadas; Clop: Cipriano Lopes; Quix: Quixabinha; Jen: Jenipapeiro; JDua: João Duarte.
